# A preconditioning nerve lesion inhibits mechanical pain hypersensitivity following subsequent neuropathic injury

**DOI:** 10.1186/1744-8069-7-1

**Published:** 2011-01-05

**Authors:** Gila Moalem-Taylor, Man Li, Haydn N Allbutt, Ann Wu, David J Tracey

**Affiliations:** 1 School of Medical Sciences, University of New South Wales, Sydney, NSW 2052 Australia; 2 Department of Neurobiology, Tongji Medical College, Huazhong University of Science and Technology, Wuhan 430030, PR China; 3 Department of Physiology, University of Sydney, NSW 2006 Australia

## Abstract

**Background:**

A preconditioning stimulus can trigger a neuroprotective phenotype in the nervous system - a preconditioning nerve lesion causes a significant increase in axonal regeneration, and cerebral preconditioning protects against subsequent ischemia. We hypothesized that a preconditioning nerve lesion induces gene/protein modifications, neuronal changes, and immune activation that may affect pain sensation following subsequent nerve injury. We examined whether a preconditioning lesion affects neuropathic pain and neuroinflammation after peripheral nerve injury.

**Results:**

We found that a preconditioning crush injury to a terminal branch of the sciatic nerve seven days before partial ligation of the sciatic nerve (PSNL; a model of neuropathic pain) induced a significant attenuation of pain hypersensitivity, particularly mechanical allodynia. A preconditioning lesion of the tibial nerve induced a long-term significant increase in paw-withdrawal threshold to mechanical stimuli and paw-withdrawal latency to thermal stimuli, after PSNL. A preconditioning lesion of the common peroneal induced a smaller but significant short-term increase in paw-withdrawal threshold to mechanical stimuli, after PSNL. There was no difference between preconditioned and unconditioned animals in neuronal damage and macrophage and T-cell infiltration into the dorsal root ganglia (DRGs) or in astrocyte and microglia activation in the spinal dorsal and ventral horns.

**Conclusions:**

These results suggest that prior exposure to a mild nerve lesion protects against adverse effects of subsequent neuropathic injury, and that this conditioning-induced inhibition of pain hypersensitivity is not dependent on neuroinflammation in DRGs and spinal cord. Identifying the underlying mechanisms may have important implications for the understanding of neuropathic pain due to nerve injury.

## Background

Peripheral nerve injury often results in neuropathic pain characterized by unpleasant and persistent increases in pain sensitivity, including hyperalgesia and allodynia. It is well recognized that nerve lesion induces neuronal and immunological changes and modifies gene and protein expression, both in the peripheral nervous system and in the spinal cord [1]. However, whether such changes affect neuropathic pain behavior following subsequent injury is not known.

Studies on axonal regeneration in the sciatic nerve have shown that a conditioning lesion of the tibial nerve (a branch of the sciatic nerve), made 2 weeks before sciatic nerve injury, causes a significant increase in axon outgrowth [2,3]. Further work has shown that regeneration of dorsal root ganglion (DRG) central processes into a peripheral nerve graft and regeneration of dorsal column fibers into a spinal cord lesion site is significantly improved by performing a peripheral nerve lesion [4-6]. Thus, a preconditioning nerve lesion appears to increase the intrinsic regenerative ability of central axons by inducing molecular changes (e.g. elevation of intracellular cyclic AMP) that allow axons to overcome myelin inhibition [7]. Studies on cerebral preconditioning have shown that a brief period of sub-lethal or mild preconditioning ischemia attenuates injury from subsequent severe ischemia [8]. This neuroprotection is achieved by promoting neuronal survival through attenuation of several injury-inducing mechanisms (e.g. excitotoxicity, ion/pH imbalance, oxidative stress, metabolic dysfunction, inflammation) and through enhancement of endogenous repair processes [9].

There are limited and conflicting data regarding the effects of an existing injury on the development of pain subsequent to a second injury. Some studies have demonstrated that noxious cutaneous stimuli elicit a powerful and long-lasting inhibition of spinal dorsal horn and trigeminal convergent neurons, termed diffuse noxious inhibitory controls (DNIC) [10,11]. DNIC effects are directly related to the duration of the conditioning painful stimulus [11] and predict inhibition of pain behaviors following prior painful insults. Indeed, activation of DNIC was confirmed in rats with chronic constriction injury of the sciatic nerve, demonstrating increased inhibition by nociceptive conditioning stimuli to the hindpaw [12]. In contrast, other studies have shown that a trigeminal nerve injury significantly accelerated the development of mechanical allodynia and hyperalgesia following a chronic constriction injury of the sciatic nerve as a second injury (day 7), in Lewis but not Sprague-Dawley or Sabra rats [13]. Repeated injury to the lumbar nerve roots at 42 days also produced enhanced mechanical allodynia and spinal neuroinflammation [14]. Thus, it appears that modulation of pain following a primary injury is greatly dependent on the location and timing of injuries, the nature of stimulus, and the strain of animals.

Here, using a rat model of neuropathic pain we investigated whether a preconditioning nerve lesion influences pain sensation and neuroinflammation following a subsequent distant peripheral nerve injury involving the same dorsal root ganglia.

## Results

### A preconditioning nerve lesion alters pain behaviors following neuropathic injury

To study the effects of preconditioning on neuropathic pain, we carried out a crush injury to one of the terminal branches of the sciatic nerve or exposed the nerve branch without crush injury (sham control), 1 week before partial ligation of the sciatic nerve (PSNL; a model of neuropathic pain) and measured pain behaviors for 30 days. The following groups of rats (n = 6 animals per group) were used: (1) Left tibial nerve crush injury 1 week before left PSNL; (2) Left common peroneal nerve crush injury 1 week before left PSNL; (3) Right tibial nerve crush injury 1 week before left PSNL. Each of these preconditioned groups was compared to a control group with a relevant sham crush 1 week before left PSNL (unconditioned).

**Figure 1:**
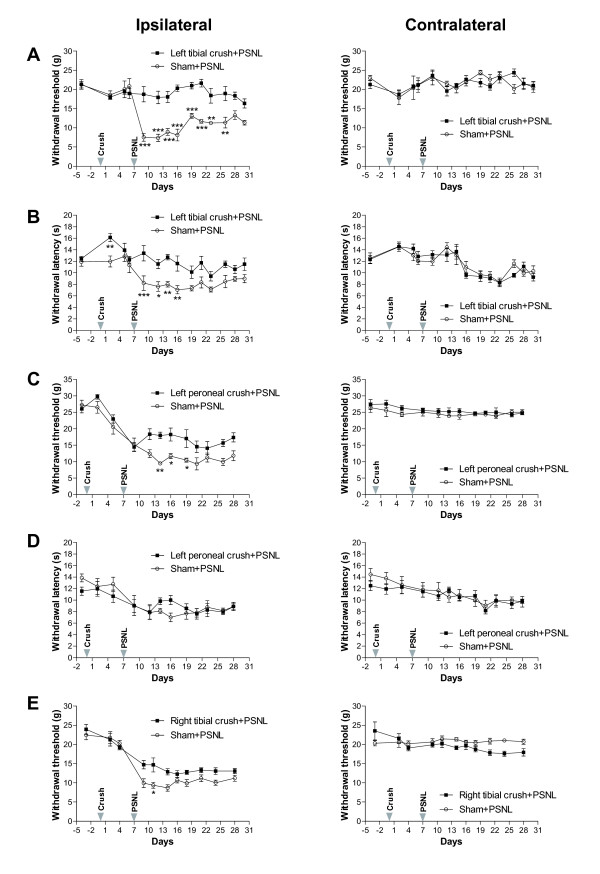
**Effects of preconditioning nerve lesion on neuropathic pain behaviors due to partial ligation of the left sciatic nerve**. **(A-B) **Tibial nerve crush injury (left) inhibits PSNL-induced mechanical and thermal pain hypersensitivity. Withdrawal thresholds to mechanical stimuli (d9-d26) **(A) **and withdrawal latencies to thermal stimuli (d9-d16) **(B) **were significantly greater in the ipsilateral left hindpaws of rats that underwent left tibial crush injury 1 week before PSNL than in rats that underwent only PSNL. No significant differences in paw withdrawal thresholds **(A) **and in paw withdrawal latencies **(B) **were observed in the contralateral right side. **(C-D) **Peroneal nerve crush injury (left) inhibits PSNL-induced mechanical, but not thermal, pain hypersensitivity. Withdrawal thresholds to mechanical stimuli (d14-d19) **(C) **were significantly greater in ipsilateral left hindpaws of rats that underwent left peroneal crush injury 1 week before PSNL than in rats that underwent only PSNL. No significant differences in paw withdrawal thresholds were observed in the contralateral right side. **(D) **No significant differences were observed in paw withdrawal latencies to thermal stimuli in both the ipsilateral and contralateral sides. **(E) **Tibial nerve crush injury (right; on the other side of the PSNL) transiently inhibits PSNL-induced mechanical pain hypersensitivity. Withdrawal thresholds to mechanical stimuli (d11) were significantly greater in ipsilateral left hindpaws of rats that underwent right tibial crush injury 1 week before PSNL than in rats that underwent only PSNL. No significant differences were observed in the right side, contralateral to PSNL. (n = 6 rats per group, *P < 0.05, ** *P *< 0.01, *** *P *< 0.001, two-way RM ANOVA followed by Bonferroni post-tests). Data are expressed as mean ± s.e.m. Arrowheads indicate day of surgery for crush injury and PSNL.

Following PSNL, all unconditioned rats developed pain hypersensitivity in the paw ipsilateral to PSNL compared with either the contralateral side or baseline values before surgery, as indicated by a sharp decrease in paw withdrawal threshold to mechanical stimuli and paw withdrawal latency to thermal stimuli (Figure 1). On the contralateral side to PSNL (right hindpaws), there were no significant differences in either the mechanical or the thermal pain sensitivity between the preconditioned (crush-injured) and unconditioned (no crush) rats (Figure 1 right panel). However, on the ipsilateral side to PSNL (left hindpaws), a preconditioning lesion of the left tibial nerve induced a long-term significant attenuation of both ligation-induced mechanical allodynia (Figure 1A) and thermal hyperalgesia (Figure 1B). Compared with unconditioned rats, preconditioned rats had significantly higher (1.6-2.5-fold; *P *< 0.01-*P *< 0.001) paw withdrawal thresholds to mechanical stimuli 2-19 days post-PSNL (Figure 1A) and significantly higher (1.5-1.6-fold; *P *< 0.05-*P *< 0.001) paw withdrawal latencies to thermal stimuli 2-9 days post-PSNL (Figure 1B). A preconditioning lesion of the left common peroneal induced a smaller but significant short-term attenuation of mechanical allodynia (Figure 1C), but not thermal hyperalgesia (Figure 1D). Compared with unconditioned rats, preconditioned (left peroneal) rats had significantly higher (1.6-1.9-fold; *P *< 0.05-*P *< 0.01) paw withdrawal thresholds to mechanical stimuli 7-12 days post-PSNL (Figure 1C). These preconditioned (left peroneal) rats also had higher (1.2-1.4-fold) paw withdrawal latencies to thermal stimuli 7-9 days post-PSNL (Figure 1D) compared with unconditioned rats, but this effect was not statistically significant. A preconditioning lesion of the right tibial nerve induced a small decrease in paw withdrawal thresholds to mechanical stimuli in the contralateral side to PSNL, though these changes were not significant (Figure 1E, right). On the ipsilateral side, a short-term attenuation of mechanical allodynia was observed, and preconditioned (right tibial) rats had a significantly higher (1.6-fold; *P *< 0.05) paw withdrawal threshold to mechanical stimuli 4 days post-PSNL (Figure 1E). There were no differences in thermal pain sensitivity between unconditioned and preconditioned (right tibial) rats on either the ipsilateral or the contralateral sides to PSNL (data not shown).

### A preconditioning nerve lesion does not affect PSNL-induced neuroinflammation in DRG and spinal cord

**Figure 2:**
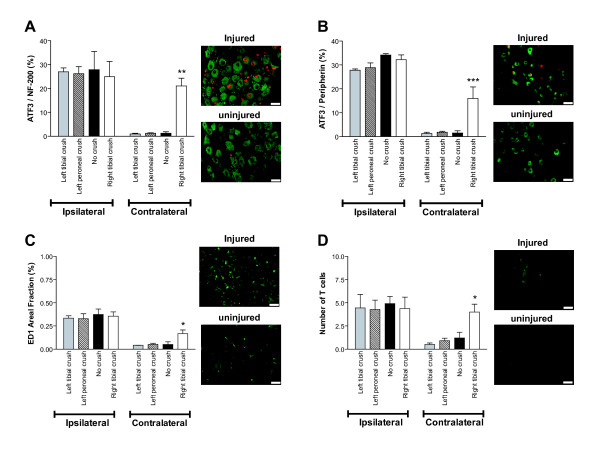
**Effects of preconditioning nerve lesion on PSNL-induced neuronal damage and inflammation in L4/5 DRGs**. **(A-B) **Percentage of ATF3+ DRG neurons in populations of large NF-200-expressing neurons, and small peripherin-expressing neurons. Compared to the contralateral uninjured side, a large increase in NF-200 neurons (green) containing ATF3+ nuclei (red) **(A) **and in peripherin positive neurons (green) containing ATF3+ nuclei (red) **(B) **was observed on the side ipsilateral to PSNL in all groups. On the ipsilateral side, there was no significant difference between preconditioned (crush-injured) and unconditioned (no crush) groups. On the contralateral side, the percentage of NF-200 neurons containing ATF3+ nuclei **(A) **and peripherin neurons containing ATF3+ nuclei **(B) **was significantly higher in the rats that underwent right tibial nerve crush injury as compared to all other groups. **(C-D) **Macrophage and T-cell presence in DRGs. Compared to the contralateral uninjured side, ED1 immunoreactivity (in green) **(C) **and the number of T cells (in green) **(D) **were markedly increased after PSNL on the ipsilateral side, but with no significant difference between preconditioned and unconditioned groups. On the contralateral side, ED1 immunoreactivity **(C) **and T-cell numbers **(D) **were significantly higher in the rats that underwent right tibial nerve crush injury as compared to all other groups. Micrographs on the right of each histogram show representative examples of immunoreactivity in DRGs from injured (ipsilateral) and uninjured (contralateral) sides. (n = 3 rats per group, *P < 0.05, ** *P *< 0.01, *** *P *< 0.001, two-way ANOVA followed by Bonferroni post-tests). Data are expressed as mean ± s.e.m. Scale bars represent 50 μm.

We next examined whether a preconditioning nerve lesion affects neuroinflammation 7 days after PSNL (14 days after crush injury) in L4/5 DRGs and in L4-6 spinal cord segments using immunohistochemistry (n = 3 animals per group). Cryosections of excised DRGs were stained for macrophages (ED1 marker) and T cells (T-cell receptor marker) [15], and for damaged neurons expressing activating transcription factor 3 (ATF3) in subpopulations of medium- to large-sized DRG neurons expressing neurofilament-200 (NF-200) [16] and small-sized DRG neurons expressing peripherin, a marker found predominantly in small sensory ganglion cells with unmyelinated C-fiber axons [17]. Cryosections of lumbar spinal cords were stained for microglia (ionized calcium binding adaptor molecule 1; IBA1 marker) and astrocytes (glial fibrillary acidic protein; GFAP marker). We found that compared to the contralateral uninjured side, PSNL induced a large increase in the level of ATF3/NF-200 (Figure 2A) and ATF3/peripherin (Figure 2B) with about 25-30% of NF-200 neurons containing ATF+ nuclei and about 28-34% of peripherin neurons containing ATF+ nuclei in the DRGs ipsilateral to the PSNL (left side). PSNL also induced a large increase in ED1 (Figure 2C) and T-cell receptor (Figure 2D) immunoreactivity in the ipsilateral DRGs, compared to contralateral uninjured DRGs. Interestingly, there were no differences between preconditioned and unconditioned rats in the ipsilateral DRGs; the preconditioning injury did not affect the percentage of large NF-200 positive neurons (Figure 2A) and small peripherin positive neurons (Figure 2B) expressing ATF3, or the density of macrophages (Figure 2C) and number of infiltrating T cells (Figure 2D) following the PSNL. On the right side, however, crush injury of the right tibial nerve by itself caused a significant increase (*P *< 0.01-*P *< 0.001) in large and small ATF3+ neurons (Figure 2A, B) and a significant increase (*P *< 0.05) in the number of macrophages and T cells (Figure 2C, D).

**Figure 3:**
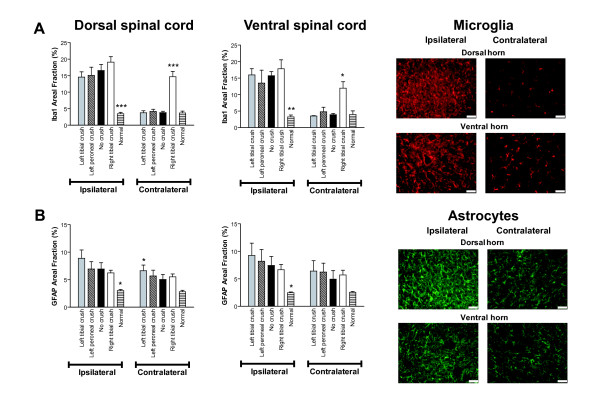
**Effects of preconditioning nerve lesion on glial activation in lumbar spinal cord (L4-6), 1 week after partial ligation of the left sciatic nerve**. **(A) **Activation of microglia in the ipsilateral dorsal and ventral horn of the spinal cord was significantly increased 7 days after PSNL in both preconditioned (crush-injured) and unconditioned (no crush) rats as compared to normal rats and to the contralateral uninjured side. Activation of microglia in the contralateral dorsal and ventral horn of the spinal cord was significantly increased only in the rats that underwent (2 weeks before) right tibial nerve crush injury. **(B) **Activation of astrocytes in the ipsilateral dorsal horn of the spinal cord was significantly increased 7 days after PSNL in both preconditioned (crush-injured) and unconditioned (no crush) groups as compared to normal rats. Astrocyte activation was significantly increased in the ventral horn of preconditioned rats (left tibial and left peroneal crush) as compared to normal rats. In the contralateral spinal cord, no significant differences were observed between the groups, except for the dorsal horn of preconditioned rats (left tibial crush) as compared to normal rats. Micrographs (right panel) show examples of immunoreactivity to IBA1 (microglia, in red) and GFAP (astrocytes, in green) in sides ipsilateral (left) and contralateral (right) to PSNL in both dorsal and ventral spinal cords of unconditioned rats. (n = 3 rats per group, *P < 0.05, ** *P *< 0.01, *** *P *< 0.001, two-way ANOVA followed by Bonferroni post-tests). Data are expressed as mean ± s.e.m. Scale bars represent 50 μm.

In the spinal cord, a considerable increase in IBA1 immunoreactivity (microglia) was observed after PSNL on the ipsilateral side of both the dorsal and the ventral horns, as compared to the contralateral uninjured side and normal controls (Figure 3A). A large increase in GFAP immunoreactivity (astrocytes) was also observed after PSNL on the ipsilateral side, and to a smaller extent on the contralateral side, as compared to normal animals (Figure 3B). However, although both microglia and astrocytes were significantly upregulated (*P*<0.05-*P *< 0.001) in the ipsilateral dorsal and ventral horns compared to normal controls, there were no significant differences between the preconditioned and unconditioned groups (Figure 3A, B). In the spinal cord contralateral to the PSNL, crush injury of the right tibial nerve by itself induced a significant increase in microglia activation in both dorsal (*P *< 0.001) and ventral (*P *< 0.05) horns (Figure 3A), but not astrocyte activation (Figure 3B). Thus, a preconditioning nerve lesion prior to PSNL did not change the level of ligation-induced neuronal damage, macrophage and T-cell infiltration into DRGs, or microglia and astrocyte activation in the spinal cord.

## Discussion

Our study shows that a preconditioning lesion distal to the experimental neuropathic injury (PSNL) inhibits the development of mechanical pain hypersensitivity. A left tibial nerve crush seven days before partial ligation of the left sciatic nerve inhibits the development of thermal hyperalgesia and mechanical allodynia. A crush injury of the left peroneal nerve or the right tibial nerve only transiently attenuates mechanical allodynia, but not thermal hyperalgesia induced by left PSNL. The paw withdrawal response of preconditioned rats was not compromised by the nerve crush injury as all rats were capable of withdrawing their hindpaw in response to mechanical or thermal stimuli. Furthermore, compared to sham operation, crush injury of the right tibial nerve by itself (Figure 1E, right panel) induced a slight reduction in paw withdrawal threshold to mechanical stimuli during the course of the experiment. It should be noted that combined crush injury of both the tibial and peroneal nerves has previously been shown to induce mechanical hypersensitivity of the paw [18].

Since the greatest effect of nerve crush on ligation-induced neuropathic pain occurred following left tibial nerve lesion, it is likely that some of the effect is due to denervation of sensory afferents in the area of the mid-plantar surface of the paw tested, an area corresponding to the cutaneous innervation of the tibial nerve [18]. However, a recent study has demonstrated that even after total tibial nerve axotomy, some evoked pain behaviors are observed in the tibial-innervated skin [19]. In addition, crush injury of the common peroneal nerve predominantly innervating the lateral half of the hairy skin of the paw [20] and crush injury of the right tibial nerve innervating the right hindpaw, also had an effect in our study. These results indicate the involvement of either systemic or central mechanisms in the preconditioning-induced pain inhibition.

The mechanisms responsible for the behavioral effects of preconditioning on neuropathic pain (Figure 1) are currently unclear. Potential mechanisms include modulation of endogenous pain controls following the initial injury, such as induction of DNIC involving inhibition of dorsal nociceptive neurons [12] or enhancement of descending net inhibition from supraspinal structures as seen following peripheral inflammation [21]. The preconditioning injury may trigger adaptive responses in resident cells within the nervous system by inducing changes in gene expression and/or post-translational modifications of existing proteins [22]. Some of these changes may serve to protect the tissue by stabilizing cell energy and protein metabolism, ameliorating the action of harmful mediators (e.g. glutamate, nitric oxide) and modifying post-injury inflammation, thereby reducing pain following the subsequent injury. Other possibilities include differential modulation of endogenous opioids in the spinal cord [23] of preconditioned and unconditioned animals, and generation of an unidentified endogenous analgesic substance, which enters the blood stream and provokes a systemic protective response after the preconditioning injury. Our finding of differential pain behaviors following the different preconditioning nerve lesions suggests that the nerve size, the type of fibers in the crushed nerve, and the extent of sensory afferent denervation influence the level of pain inhibition. It is likely that several mechanisms are involved.

We found no difference in PSNL-induced neuronal damage and macrophage and T-cell infiltration into the DRGs, and no difference in PSNL-induced astrocyte and microglia activation in the spinal cords of preconditioned and unconditioned animals. Interestingly, crush injury of the tibial nerve by itself induced significant neuronal damage and neuroimmune activation, and the subsequent PSNL did not have a substantial additive effect (Figure 2, 3). Although there were no overall differences in the magnitude of the neuroinflammatory response between the study groups, there might be differences in the phenotype (e.g., cytokine profile) of the cells involved. Activation of spinal microglia and astrocytes and concomitant release of proinflammatory products are strongly implicated in pathological neuropathic pain [24-26]. Microglia cells, in particular, are emerging as critical players in peripheral injury-induced neuropathic pain [27]. In contrast, our data suggest that the general level of spinal glia activation is not necessarily correlated with the level of pain sensitivity. In support of this, previous studies have demonstrated that neuropathic pain behaviors preceded and did not strictly correlate with microglial responses following peripheral nerve injury [28]. Additionally, two different models of cancer pain exhibiting pain hypersensitivity resulted in severe spinal astrogliosis without activation of microglia [29]. It has been suggested that microglia do not comprise a single, uniform cell population, but rather cells with diverse phenotypes and that microglial commitment to a phenotype can be changed depending on the nature of stimuli [30]. Thus, there might be a distinct pattern of glial activation that confers a beneficial neuroprotective effect as opposed to a detrimental excitotoxic effect [30] following peripheral nerve injury preceded by preconditioning. Indeed, glial activation in the nervous system has been shown in some instances to be neuroprotective by release of anti-inflammatory factors and by protection against neuronal damage [24,31].

## Conclusions

In summary, our results show that pain sensation might be affected by a prior injury. The mechanisms underlying the conditioning-induced attenuation of pain hypersensitivity following neuropathic injury are not known. It is logical to assume that the long-lasting protective effect by the preconditioning tibial nerve crush injury is at least partially due to denervation of the skin examined by the sensory tests employed in the present study. However, the transient inhibition of mechanical allodynia by the preconditioning crush injury of the peroneal nerve, and the tibial nerve on the other side of the PSNL, suggests an involvement of systemic and/or central changes in the preconditioned animals. Identifying the underlying mechanisms may have important implications for the understanding of persistent pain that develops after nerve injury and for developing treatment approaches to ease neuropathic pain.

## Methods

### Animals

Inbred male Wistar rats (Biological Resources Centre, University of New South Wales, Australia), 7-8 weeks of age at the commencement of study were used. Animals were housed at approximately 22°C in groups of six under a 12-h light/dark cycle with free access to food and water. Protocols were approved by the Animal Care and Ethics Committee of the University of New South Wales and adhered to the guidelines of the Committee for Research and Ethical Issues of the International Association for the Study of Pain.

### Nerve crush injury

Animals were anesthetized with halothane in a 1:1 mixture of O_2 _and N_2_O and the sciatic nerve was exposed distally with its three terminal branches: the sural, common peroneal and tibial nerves. The tibial or peroneal nerve was crushed for 30 seconds by a pair of fine forceps with a smooth flat crushing surface. The crush injury resulted in a flattened and transparent section of nerve at the crush area. Sham controls involved exposure of the relevant nerve without any lesion. Muscle was closed in layers by suturing and the skin clipped.

### Partial ligation of the sciatic nerve

The surgical procedure was based on that described by Seltzer et al [32]. Rats were anesthetized with halothane in a 1:1 mixture of O_2 _and N_2_O. An incision was made at the proximal thigh and the left sciatic nerve exposed. About one third of the diameter of the left sciatic nerve was tightly ligated just proximal to its branch to the posterior biceps and semitendinosus muscles, using 7-0 silk (Tyco Healthcare, Norwalk, CT, USA). A sham operation was carried out on the right hind limb of each animal, in which the sciatic nerve was exposed but not damaged in any way. Muscle layers were closed with 4-0 silk sutures and the skin wounds closed with skin staples.

### Behavioral testing

Rats were habituated to the behavioral testing apparatus for 30 min to 1 hr prior to data collection and the testing environment was kept quiet and well controlled. Behavioral tests were performed 3 times a week for 30 days, with a baseline measurement before surgery. Thermal hyperalgesia was assessed as previously described [33], by exposing the mid-plantar surface of the hindpaw to a beam of radiant heat through a transparent glass surface using a plantar analgesia meter for paw stimulation (Ugo Basile, Italy). The latency of withdrawal from the heat stimulus was automatically recorded as the time taken from the onset of radiant heat stimulation to withdrawal of the paw. A cut-off latency of 22 sec was pre-set to prevent tissue damage. Mechanical allodynia was assessed by placing an animal on an elevated wire grid and stimulating the plantar surface of the hindpaw, using an electronic von Frey anesthesiometer (IITC Inc., Woodland Hills, CA, USA). The probe was gently applied to the centre of the paw just posterior to the paw pads with slowly increasing force until the rat withdrew its paw in response to the stimulus. The device automatically recorded and displayed the force (in grams) that elicited a withdrawal response. Thermal latencies and mechanical thresholds were measured four times for each paw, with a 3-5 min interval between measurements and the mean was calculated. All behavioral experiments were repeated twice by different experimenters.

### Immunohistochemistry

Seven days after partial ligation of the sciatic nerve, rats were anesthetized using an overdose of sodium pentobarbitone (120 mg/kg i.p.). They were then perfused through the aorta with 0.9% saline containing heparin followed by fresh 4% paraformaldehyde in 0.1 M phosphate buffer (pH 7.4) for tissue fixation. Both left and right L4 and L5 DRGs as well as L4-L6 lumbar spinal cord segments were harvested. Tissues were post-fixed in 4% paraformaldehyde for 6 h and then transferred to 30% sucrose overnight. Cryosections (10-20 μm thick) were prepared and stained as previously described [34]. DRG sections were stained for T cells with mouse anti-rat monoclonal antibody to αβ T-cell receptor, clone R73 (1:200; BD Biosciences-PharMingen, San Diego, CA, USA) and for macrophages with mouse anti-rat CD68, clone ED1 (1:250; Serotec, Oxford, UK). Double labeling was performed with rabbit anti-ATF3 (1:400; Santa Cruz Biotechnology, Santa Cruz, CA, USA) and either mouse anti-NF-200 (1:500; Sigma, Castle Hill, New South Wales, Australia) or mouse anti-peripherin (1:400; Chemicon International, Billerica, MA, USA). Spinal cord sections were stained for microglia with rabbit anti-IBA1 (1:2000; Wako, Osaka, Japan) and for astrocytes with mouse anti-GFAP (1:2000; Chemicon International). Sections were blocked and then incubated with the primary antibody. Elimination of the primary antibody was used as a negative control. The sections were washed 4 times and incubated with a secondary antibody as appropriate: donkey anti-mouse IgG conjugated with Cy2 (1:100; Jackson ImmunoResearch, West Grove, PA, USA) or donkey anti-rabbit IgG conjugated with Cy3 (1:400; Jackson ImmunoResearch). In the case of double labeling, both secondary antibodies were used. Sections were washed 4 times and treated with fluorescent mounting medium (DakoCytomation) before being cover-slipped.

### Image analysis

Sections were viewed on an Olympus fluorescence microscope. Images were captured using an Olympus DP70 camera and DP Controller software (Olympus Tokyo, Japan) and were taken from 3-6 sections in each animal. In each photograph, immunoreactivity was evaluated using NIH ImageJ software (NIH, Bethesda, USA) in two ways: (i) for immunoreactivity that contained clear cellular staining, single stained cells (αβ T-cell receptor) and doubled-stained cells (ATF3/NF-200 or ATF3/peripherin) were counted manually using the cell counter plug-in; (ii) for immunoreactivity where individual cells were difficult to demarcate, thresholding was used to detect labeled structures and the % areal fraction covered by stained structures determined. In the spinal cord, regions of both dorsal and ventral horns were quantified in areas of the sciatic territories [35]. The images were taken using a 40X objective lens. Each field of view measured 442 × 333 μm.

### Statistical analysis

All data are presented as mean ± s.e.m. Immunohistochemistry data were analyzed with a two-way analysis of variance (ANOVA) followed by Bonferroni post-tests with animal group and area (ipsilateral/contralateral) as factors. Behavioral data were analyzed with repeated measures two-way ANOVA followed by Bonferroni post-tests with animal group and time (days) as factors. A probability of 0.05 or less was considered statistically significant.

## Abbreviations

DRG: dorsal root ganglion; DNIC: diffuse noxious inhibitory controls; PSNL: partial ligation of the sciatic nerve; ATF3: activating transcription factor 3; NF-200: neurofilament-200; IBA1: ionized calcium binding adaptor molecule 1; GFAP: glial fibrillary acidic protein; ANOVA: analysis of variance.

## Competing interests

The authors declare that they have no competing interests.

## Authors' contributions

GM-T designed and coordinated the study, carried out some experiments, analyzed data and wrote the manuscript. ML and HNA carried out some behavioral testing, tissue processing and immunohistochemical analysis. AW and DJT participated in study design and data analysis. All authors read and approved the final manuscript.
